# Bayesian evaluation of the performance of three diagnostic tests for *Plasmodium falciparum* infection in a low-transmission setting in Kilifi County, Kenya

**DOI:** 10.12688/wellcomeopenres.15204.3

**Published:** 2019-10-01

**Authors:** Marshal M. Mweu, Juliana Wambua, Fixtan Njuga, Philip Bejon, Daniel Mwanga

**Affiliations:** 1 School of Public Health, University of Nairobi, Nairobi, Kenya; 2 KEMRI-Wellcome Trust Research Programme, Kilifi, Kenya; 3 Centre for Tropical Medicine and Global Health, Nuffield Department of Clinical Medicine, University of Oxford, Oxford, UK

**Keywords:** Bayesian latent class model, Plasmodium falciparum, Light microscopy, Rapid diagnostic test, PCR, Test evaluation

## Abstract

**Background:** Central to the successful elimination of *Plasmodium falciparum* malaria, are tests with superior capability of diagnosing low-density parasitaemias. Empirical evidence on the performance of the commonly available diagnostics (light microscopy (LM), rapid diagnostic tests (RDT) and polymerase chain reaction (PCR)) is needed to better inform case management and surveillance activities within primary health care settings where elimination of *falciparum* malaria is targeted. The objective of this study was to estimate the sensitivity (Se) and specificity (Sp) and predictive values of LM, RDT and PCR tests for *P. falciparum* infection in children, while evaluating the effect of specific covariates on the accuracy of the tests.

**Methods:** The study enrolled 1,563 children presenting with fever (axillary temperature ≥ 37.5 ^0^C) to the Ngerenya dispensary, Kilifi County between March and December 2014. A Bayesian latent class model (BLCM) was fitted to the participants’ diagnostic data obtained from blood samples that were screened for the presence of *P. falciparum* using the three tests.

**Results:** The PCR assay registered a higher Se (97.6% [92.0; 99.7]) than LM (84.0% [74.8; 91.0]) but similar to RDT (92.2% [84.4; 97.0]). However, the assay showed a similar Sp (98.9% [98.2; 99.4]) to both RDT (99.4% [98.9; 99.7]) and LM (99.5% [99.0; 99.8]). Regarding predictive values, the tests yielded statistically similar estimates of positive and negative predictive values (PPV and NPV). A serial interpretation of the results of RDT and LM raised the PPVs and NPVs to >98%.

**Conclusions:** LM and RDT afford high Se and Sp in symptomatic care-seeking children in this low *P. falciparum* prevalence setting. A serial combination of the tests assures high PPV and NPV estimates. These elements, coupled with the wide deployment and affordability of the tests, lend the tests useful for guiding clinical care and surveillance activities for *P. falciparum* within elimination settings.

## Introduction

Malaria persists as a leading cause of morbidity and mortality globally ^1^ and in Kenya, close to 70% of the population is at risk of the disease. *Plasmodium falciparum* is the most preponderant malaria parasite in the country associated with over 99% of malaria infections ^2^. However, studies report a declining trend of *P. falciparum* prevalence particularly along the Kenyan coastal region ^3, 4^.

The Kenya national guidelines for diagnosis and treatment of malaria dictate that malaria treatment should be informed by parasitological diagnosis ^5^. Light microscopic (LM) examination of thin or thick blood smears is held as the standard method for malaria diagnosis ^5^. The test is inexpensive, generally exhibits high sensitivity (Se) and specificity (Sp) and permits parasite quantification which is a vital attribute in evaluating disease severity and guiding appropriate therapy ^6^. Nevertheless, the test is fraught with some challenges: it requires good lab equipment and well trained microscopists that are often lacking in poor settings and it may display poor Se especially in cases of low parasitaemia given its detection limit of approximately 50 – 100 parasites per µl of blood under field conditions ^6^.

Rapid diagnostic tests (RDTs) are immunochromatographic tests that detect specific parasite antigens ^7^: the *P. falciparum*-specific histidine-rich protein II (HRP–II) or lactate dehydrogenase (LDH) and *Plasmodium* aldolase, which is pan-specific ^8^. RDTs are hailed for their rapidity, usage simplicity, suitability for use in remote settings with limited equipment and trained staff and display high Se and Sp under field conditions ^9^. However, their Se may be limited in situations of low parasitaemia ^10^.

With declining *P. falciparum* transmission rates and thus its prevalence within the country ^3, 11^, low parasite densities in the population are anticipated that may compromise the Se of LM and RDTs ^9^. This situation may warrant alternative tests capable of detecting diminished levels of parasitaemia. Molecular-based techniques such as polymerase chain reaction (PCR) assays are touted as being less subjective and affording high Se and Sp in low parasite density settings ^12, 13^. Nevertheless, since PCRs may detect non-viable parasites – quite sensibly common in elimination settings – their utility in guiding national guidelines for clinical case management is vague. Empirical evidence on the performance of these diagnostics in low-transmission settings is thus necessary to better inform management and surveillance efforts for *P. falciparum* malaria.

The diagnostic performance of RDTs has previously been evaluated using LM/PCR as a reference test ^14– 18^. A drawback of this approach is that, given the imperfection of these references, the index tests’ characteristics are subject to bias. Moreover, for index tests presumed to have superior accuracy to the existing reference test, their evaluation based on the reference test is impractical. In the absence of a reliable reference test, latent class models (LCMs) allow for the simultaneous estimation of Se and Sp of two or more tests without any assumption about the underlying true disease status of each individual ^19^. LCMs can be fit using either maximum likelihood or Bayesian methods ^20^. Essentially, Bayesian methods are preferable when observed data are insufficient ^21^.

In heterogeneous populations, when information on some covariates perceived to influence the performance of index tests is available, stratified estimates of Se and Sp are computable and, reasonably, are more relevant than singular estimates. Of note, estimates of Se and Sp are characteristics specific to a test. However, once the test is applied in any given population, our interest rests on predictive values since the present concern is whether a particular tested individual has/does not have the disease in question given his/her test status. Therefore, the objective of this study was to estimate (within a Bayesian framework) the Se and Sp and predictive values of LM, RDT and PCR diagnostic tests for *P. falciparum* infection in children, while evaluating the effect of specific covariates on the performance of the tests.

## Methods

### Study area and population

The study participants comprised children aged <15 years presenting with fever (axillary temperature ≥ 37.5°C) to the Ngerenya dispensary, Kilifi County between March and December, 2014. This facility is situated within the Kilifi Health and Demographic Surveillance System (KHDSS) area on the Kenyan coast where, among other infectious diseases, malaria screening is conducted routinely ^22^. Notably, the KHDSS area spans a population of approximately 260,000 persons in an area of about 891km ^2^ ^22^. The area experiences a bimodal rainfall pattern, with long rains coming in the months of April to July and short rains in October and November. Malaria is endemic and transmission occurs throughout the year, with annual entomological inoculation rates ranging between 30 and 100 ^2^.

### Sample collection

At the dispensary, blood samples were collected from the study participants for immunological examinations and assessment of *P. falciparum* infections. Specifically, a 50 µl finger prick blood sample was collected for RDT, a slide for LM and an EDTA for PCR. Rapid diagnosis was carried out by trained health workers at the facility while slide and EDTA blood samples were stored in cooler boxes and transported to the KEMRI-Wellcome Trust Research laboratory, Kilifi where the LM and PCR tests were run by trained laboratorians. Examiners for parasitaemia using any of the diagnostics were blinded to the results of the other tests. Besides the samples, the participants’ socio-demographic characteristics (area of residence, age, sex and visit date) were captured.

### Ethical considerations

Parents/guardians of enrolled children provided written informed consent agreeing to their children’s participation in the study. In addition, assent was secured from the participating minors. Approval for the study was obtained from the Kenya Medical Research Institute (KEMRI) Ethical Review Committee (SSC No. 2617).

### Target condition

The latent (unobserved) infection status (referred to here as parasitaemia) targeted for detection by the three tests: LM, RDT and PCR, represents a blood sample containing either the live *P. falciparum* parasite or its HRP-II antigens/products or debris at any concentration level.

### Light microscopy (LM)

Malaria microscopy was performed as per standard guidelines ^23^. Briefly, thick and thin blood films were stained in 3% Giemsa solution for 45 to 60 minutes and examined under a light microscope at 1000× magnification for malaria parasites. Parasite quantification was achieved by counting the number of malaria parasites per 200 leucocytes. Parasite density per µl of blood was estimated assuming 8000 leucocytes per µl of blood and reported by species i.e. *P. falciparum*, *Plasmodium malariae* and *Plasmodium ovale*. For analytical purposes, *P. falciparum* densities above zero constituted a positive result; otherwise negative.

### Rapid diagnostic test (RDT)

A CareStart™ Malaria RDT kit (Cat No. G0141, AccessBio Inc.) was used to test for the presence of *P. falciparum* specific HRP-II antigens in the collected blood samples in accordance with instructions contained in the CareStart™ Malaria manual (AccessBio). A total of 5 µl of blood was added into sample wells followed by 60 µl of assay buffer solution added to assay wells. The blood-buffer mixture was then allowed to flow towards the test and control windows. The presence of two colour bands denoted a positive result; one band (the control line) indicating a negative result.

### Polymerase chain reaction (PCR) assay

The PCR analysis was conducted as described elsewhere ^24, 25^. Briefly, 30 µl of blood was used for DNA extraction using a QIAxtractor machine (QIAGEN, Hilden, Germany). In a subsequent step, the extracted DNA was eluted in a volume of 100 µl, after which 5 µl of sample DNA was amplified in a thermal cycler (Applied Biosystems *™* 7500 Real *-* Time PCR System, Applied Biosystems, Foster City, CA). Specifically, 5 µl of DNA was added to 45 µl of amplification mixture containing TaqMan buffer, 250 µM of each deoxynucleotide triphosphate, 0.125 U Amplitaq Gold polymerase, oligonucleotide primers and TaqMan probe (5’-FAM-AACAATTGGAGGGCAAG-NFQ-MGB-3’) ^24^. In about 10 minutes at 95°C pre-incubation, PCR amplification was carried out for 45 cycles (15s 95°C, 1 min 60°C) using a TaqMan assay for the highly conserved *P. falciparum* multicopy 18S ribosomal RNA gene. Quantification was achieved based on the Applied Biosystems 7500 software v2.0.6. The method has a quantification limit of 4.5 parasites/µl of blood. Three negative control wells and seven serial dilutions of DNA extracted from an *in vitro* culture of the *P. falciparum* 3D7 parasite strain were included on each plate as standards ^24^. Samples with PCR readings beyond zero were considered positive.

### Population classification

Organisationally, within the Kenyan health system, a dispensary denotes a primary care unit serving an immediate catchment population ^26^. Arguably, therefore, the facility data represented a sample drawn from a single target population that formed the basis for derivation of Se and Sp of the three tests.

### Statistical analysis

Initially, based on the participants’ visit dates, a dichotomous ‘season’ variable (‘wet’: [April – July, October and November] versus ‘dry’: [March, August, September, December]) was generated. A Bayesian latent class model (BLCM) built in OpenBUGS v3.2.2 ^27^ but called from R software v3.4.3 via the ‘ BRugs’ package ^28^ v0.9-0 was used to infer prevalence, the tests’ characteristics and corresponding predictive values. Notably, the analysis was informed by the guideline for standards for reporting diagnostic accuracy studies that use BLCMs (STARD-BLCM) ^29^. Code used to run this analysis is available as underlying data ^30^.

In fitting a BLCM, three assumptions are necessary: (1) the target population should consist of two or more subpopulations with different prevalences, (2) the Se and Sp of the tests under evaluation should be constant across the subpopulations and (3) the tests should be conditionally independent given the disease status ^19^. For our situation, the three tests were assumed to be conditionally independent given an individual’s *P. falciparum* infection status. This is sensible considering that the tests target different aspects of the parasite. As such, statistically, for an individual whose infection status is known, their probability of testing positive (or negative) to one of the tests remains the same regardless of their prior outcomes to the other tests. In order to evaluate the separate effects of ‘season’, ‘sex’ and ‘age’ (dichotomised into <5 yrs and ≥ 5yrs ^31^) on the Se and Sp estimates of the tests, we relaxed the assumption on constancy of the tests characteristics by stratifying the single population into subpopulations defined by the levels of the covariates. This allowed for the computation of stratum-specific tests estimates. Hypotheses for the differences between the stratified estimates were evaluated using a Bayesian *P* – value.

Counts ( *O _k_*) of the different test combinations (e.g. +,+,+) were assumed to follow a multinomial distribution of the form:

$$O_{k}|Se_{ik}Sp_{ik}P_{k}∼multinomial\text{(}prob_{k}\text{,}\;n_{k}\text{)}$$

Where *Se _ik_* and *Se _ik_* represent the respective test characteristics for test *i* ( *i* =1,2,3) in subpopulation *k* and *p _k_* is the specific prevalence for the *k ^th^* ( *k* =1,2) subpopulation. *Prob _k_* is a vector of probabilities of observing the different combinations of test results, and *n _k_* reflects the number of individuals tested for the *k ^th^* subpopulation. For instance, in the 1 ^st^ subpopulation for an individual testing positive to each of the three tests, *prob*_1_ is given by:

$$\begin{array}{l} prob_{\text{1}}=Pr\text{(}T_{\text{1}}^{\text{+}}T_{\text{2}}^{\text{+}}T_{\text{3}}^{\text{+}}|D^{+}\text{)}+Pr\text{(}T_{\text{1}}^{\text{+}}T_{\text{2}}^{\text{+}}T_{\text{3}}^{\text{+}}|D^{-}\text{)}\\ \;=Se_{\text{11}}Se_{\text{21}}Se_{\text{31}}P_{1}+\text{[1}-Sp_{\text{11}}\text{][1}-Sp_{\text{21}}\text{][1}-Sp_{\text{31}}\text{][1}-P_{\text{1}}\text{]} \end{array}$$

For each covariate, the resulting two subpopulations furnished 14 degrees of freedom sufficient to estimate 14 parameters (stratum-specific Se and Sp of the three tests and two subpopulation prevalences) – suggesting identifiability of the model. Of note, model identifiability can at least be justified if the number of subpopulations ( *k*) and tests ( *i*) fulfil the equation: *k* ≥ *i*/(2 ^*i–*1^– 1) ^32^.

Positive and negative predictive values (PPV and NPV respectively) associated with test *i* and subpopulation *k* were derived as follows:

$$\begin{array}{l} \;ppv=P_{k}Se_{ik}/(P_{k}Se_{ik}+\text{[1}-P_{k}\text{][1}-Se_{ik}\text{]})\\ npv=\text{[1}-P_{k}\text{]}Sp_{ik}/(P_{k}\text{[1}-Se_{ik}\text{]}+\text{[1}-P_{k}\text{]}Sp_{ik}) \end{array}$$

Non-informative priors ( *beta*(1,1)) were used to fit the Bayesian model since no reliable prior information was available for any of the tests parameters. A separate (non-stratified) model ignoring differences in tests Se and Sp across covariate levels was also fitted and the relative goodness of fit for the nested models compared using the Deviance Information Criterion (DIC) (the model with the smaller DIC value being more preferable).

The models were initialised with two Markov Chain Monte Carlo chains with different values. Each chain comprised 70,000 samples, with the first 20,000 being discarded as the burn-in. Convergence of the chains was evaluated by visual appraisal of the time series plots of selected variables and the Gelman-Rubin diagnostic plots. The posterior distribution of the subpopulation prevalences, the Se and Sp of the three tests, as well as the predictive values were reported as the median and the corresponding 95% posterior credible intervals (PCI).

## Results

The cross-tabulated counts of the three tests’ outcomes by covariate level are displayed in Table 1. The sample comprised 1563 children, of whom 65.8% ( *n* = 1029) were <5 years of age, 47.3% ( *n* = 739) were female and 34.5% ( *n* = 492) made visits during the dry season.

**Table 1.** Cross-classified results by stratum for rapid diagnostic test (RDT), light microscopy (LM) and polymerase chain reaction (PCR) tests for diagnosis of *P. falciparum* infection in the study population in Ngerenya, Kilifi County, Kenya.

Stratum	Tests outcomes combinations (RDT; LM; PCR)								Total (%)
	+++	++-	+-+	-++	+--	-+-	--+	---	
Single population	67	1	12	5	9	7	17	1445	1563 (100%)
Age									
<5yrs	24	1	6	2	5	4	11	976	1029 (65.8%)
≥5yrs	43	0	6	3	4	3	6	469	534 (34.2%)
Sex									
Female	27	0	4	1	2	3	9	693	739 (47.3%)
Male	40	1	8	4	7	4	8	752	824 (52.7%)
Season									
Dry	29	0	6	0	3	1	4	449	492 (34.5%)
Wet	38	1	6	5	6	6	13	996	1071 (68.5%)

The stratum-specific estimates of Se and Sp of the three tests for *P. falciparum* are presented in Table 2. There were no detectable significant differences between the covariate-stratified tests’ estimates as indicated by the Bayesian *P*-value. Furthermore, the non-stratified model gave better fit ( *DIC* =41.7) to the data than any of the covariate-stratified models ( *DICs* =[72.9; 72.0; 69.9]) and was thus utilised for subsequent analyses.

**Table 2.** Stratum-specific estimates of sensitivity and specificity of rapid diagnostic test (RDT), light microscopy (LM) and polymerase chain reaction (PCR) tests for *P. falciparum* infection and a Bayesian *P*-value for the difference between the stratified estimates.

Test parameter ^a^	Covariate		Bayesian *P*-value ^c^
	Age		
	<5yrs Estimate (95% PCI ^b^)	≥5yrs Estimate (95% PCI)	
*Se _RDT_*	89.8 (74.4; 97.7)	92.0 (81.9; 97.7)	0.38
*Se _LM_*	77.6 (60.5; 90.0)	86.3 (75.0; 94.1)	0.16
*Se _PCR_*	93.3 (79.5; 99.1)	98.4 (91.6; 99.9)	0.14
*Sp _RDT_*	99.5 (98.9; 99.9)	99.1 (97.9; 99.7)	0.81
*Sp _LM_*	99.6 (99.0; 99.9)	99.3 (98.2; 99.8)	0.76
*Sp _PCR_*	98.9 (98.1; 99.5)	98.7 (97.4; 99.6)	0.61
	Sex		
	Female Estimate (95% PCI)	Male Estimate (95% PCI)	
*Se _RDT_*	94.1 (81.2; 99.3)	89.4 (78.2; 96.2)	0.76
*Se _LM_*	84.8 (69.5; 94.5)	81.9 (69.4; 91.0)	0.63
*Se _PCR_*	97.4 (87.2; 99.9)	96.0 (87.0; 99.5)	0.63
*Sp _RDT_*	99.6 (99.0; 99.9)	99.0 (98.2; 99.6)	0.92
*Sp _LM_*	99.5 (98.8; 99.9)	99.4 (98.7; 99.8)	0.57
*Sp _PCR_*	98.7 (97.6; 99.4)	99.0 (98.0; 99.6)	0.29
	Season		
	Dry Estimate (95% PCI)	Wet Estimate (95% PCI)	
*Se _RDT_*	97.6 (87.8; 99.9)	86.8 (74.5; 94.8)	0.96
*Se _LM_*	80.7 (65.6; 91.5)	84.8 (72.3; 93.4)	0.32
*Se _PCR_*	97.6 (88.0; 99.9)	95.8 (86.3; 99.5)	0.67
*Sp _RDT_*	99.2 (98.1; 99.8)	99.4 (98.7; 99.8)	0.40
*Sp _LM_*	99.6 (98.8; 100.0)	99.4 (98.7; 99.8)	0.75
*Sp _PCR_*	99.0 (97.8; 99.7)	99.8 (97.9; 99.4)	0.67

^a^Median estimates

^b^Posterior credible interval

^c^Value is considered significant if it lies outside the range 0.025; 0.975

Results of the estimates of Se and Sp of the three tests together with their respective predictive values and prevalence of *P. falciparum* are shown in Table 3. The PCR assay recorded a higher Se (97.6; 95% PCI [92.0; 99.7]) than LM (84.0; 95% PCI [74.8; 91.0]) but similar to RDT (92.2; 95% PCI [84.4; 97.0]). Nonetheless, the assay registered a similar Sp (98.9; 95% PCI [98.2; 99.4]) to both RDT (99.4; 95% PCI [98.9; 99.7]) and LM (99.5; 95% PCI [99.0; 99.8]). As for predictive values, the tests had statistically similar estimates of PPV and NPV. Since PPVs were comparably lower than NPVs, in a bid to bolster their estimates, a serial interpretation of the results of RDT and LM led to a considerable improvement in PPV (99.9; 95% PCI [99.8;100.0]) at a negligible expense of NPV (98.7 95% PCI [97.9; 99.2]).

**Table 3.** Estimates of prevalence, sensitivity and specificity of rapid diagnostic test (RDT), light microscopy (LM) and polymerase chain reaction (PCR) tests for *P. falciparum* infection and their corresponding predictive values (negative and positive predictive values) together with the serial interpretation of the results of RDT and LM.

Parameter	Estimate (95% PCI)
*Se _RDT_*	92.2 (84.4; 97.0)
*Se _LM_*	84.0 (74.8; 91.0)
*Se _PCR_*	97.6 (92.0; 99.7)
*Sp _RDT_*	99.4 (98.9; 99.7)
*Sp _LM_*	99.5 (99.0; 99.8)
*Sp _PCR_*	98.9 (98.2; 99.4)
*P*	5.6 (4.5; 6.8)
*NPV _RDT_*	99.5 (99.0; 99.8)
*NPV _LM_*	99.1 (98.4; 99.5)
*NPV _PCR_*	99.9 (99.5; 100.0)
*PPV _RDT_*	89.4 (81.7; 94.9)
*PPV _LM_*	90.5 (82.7; 95.8)
*PPV _PCR_*	83.5 (75.1; 90.3)
*Serial _NPV_*	98.7 (97.9; 99.2)
*Serial _PPV_*	99.9 (99.8; 100.0)

## Discussion

Using latent class analysis we have estimated the accuracy of LM, RDT and PCR tests for the diagnosis of *P. falciparum* infection in children along with their associated predictive values. Enøe *et al*. ^20^ contend that a BLCM framework permits derivation of true estimates of index tests devoid of classification errors that may be introduced by the utilisation of an imperfect reference test. Thus, the findings of this study can be considered readily generalisable to other settings with similar *P. falciparum* infection burden in children.

Since malaria transmission dynamics have been shown to differ by age ^3^, season ^33^ and sex ^34^, it is conceivable that the accuracy of malaria diagnostics may be influenced by these covariates. To test for this, the Se and Sp of the three tests were allowed to vary by the aforementioned covariate levels. However, it was shown that neither of the tests’ characteristics differed significantly across the levels of any of the covariates. This implies that the performance of the tests is not influenced by either the prevailing season or the age and sex of the presenting patient. Nonetheless, in São Tomé and Príncipe, LM was reported to have a lower Se in afebrile under-five children suggesting the inadequacy of the test in detection of low-density parasitaemias ^35^.

In the present study setting, the prevalence of *P. falciparum* infection was estimated to be 5.6% [95% PCI 4.5; 6.8], suggesting a low transmissibility of the parasite in the population. Accordingly, the PCR assay registered a higher Se estimate than LM but similar to RDT upholding its capability in detection of low density infections ^10, 35– 37^. In particular, Manning *et al*. ^37^ recorded comparable Se estimates for both a nested PCR and RDT in the diagnosis of severe falciparum malaria among Papua New Guinean children. The PCR’s superiority to LM in detection of low parasitaemias is owed to its low detection limit of <5 parasites/µl of blood ^38, 39^ compared to an LM’s limit of roughly 20 parasites/µl of blood in research settings ^6^. Nonetheless, in some reference laboratories LM may detect parasite densities in the region of 10 parasites/µl ^40^. It was further shown that both LM and RDT achieved comparable Se estimates, which coincides with observations of similarity in these estimates at ~100 parasites/µl of blood ^41^.

False negative RDT results that may compromise the test’s Se estimate, are reported to occur when *P. falciparum* HRP-II genes are deleted from a large segment of the parasite population ^42^. False negativity may also arise where parasitaemic levels fall below the detection threshold – that is, 100 parasites/µl of blood. Besides, in tropical settings, extreme heat and humidity may degrade the antibodies that bind antigens resulting in negative test outcomes ^43^. As regards Sp, the three tests yielded comparable estimates that concur with findings observed elsewhere ^35, 44, 45^. In a low-prevalence setting, false positive test results (that undermine a test(s) Sp) represent a key concern. False positivity in RDTs has been noted due to cross reactions in rheumatoid factor positive patients, gametocytaemia or in situations of persistent antigenaemia with HRP-II antigens in previously treated patients ^46^. Especially due to HRP-II antigenaemia, RDTs that target the LDH antigens are evidently more suitable in monitoring treatment efficacy owing to their rapid clearance from blood ^47^. False positives by PCR may be attributable to detection of non-viable parasites (parasite debris) in treated patients or contamination in the laboratory process.

In this population, the three tests displayed comparable estimates of PPV and NPV. In particular, these estimates signify a reduced confidence in a positive compared to a negative test result ascribable to the low *P. falciparum* prevalence. A serial interpretation of the results of both RDT and LM raised the confidence to >98% in both positive and negative test outcomes. Consequently, in this low-transmission setting where false positives are increasingly expected, the most optimal testing strategy should be one that has all individuals initially screened by the more sensitive RDT test, with any resulting positives followed up with the more specific LM. Only those individuals positive to both tests should be eligible for treatment. This multiple-test approach is pivotal to reducing the risk of parasite resistance that can occur when individuals are unnecessarily subjected to artemisinin therapy ^48^. The rationale for employing the RDT-LM test combination owes to the wide deployment and affordability of the tests in most primary care settings ^49^ granting them promise towards informing clinical care and surveillance activities aimed at eliminating *falciparum* malaria. Moreover, as quantification of parasitaemias is central to the management of severe malaria and assessment of treatment response ^36^, in this respect, LM affords an added merit. By contrast, as PCRs demand hefty investment in equipment and reagents as well as highly trained personnel, their potential for routine use in low-resource field settings is limited.

The strong confidence realised in a negative test outcome is especially fundamental in a low-transmission setting where the preponderance of low-density infected individuals calls for their accurate detection not only to inform treatment but also to guide successful *P. falciparum* malaria elimination efforts. Importantly, low-density parasitaemic individuals present as potential reservoirs of infection to uninfected mosquitoes so that, if undetected, transmission may be sustained silently ^36^.

## Conclusions

Using a Bayesian approach, we have derived the Se and Sp of LM, RDT and PCR for the diagnosis of *P. falciparum* infection in symptomatic care-seeking children as well as their associated predictive values. It was shown that the PCR assay’s Se was significantly higher than that of LM but similar to RDT. Nevertheless, the Sp estimates of the three tests were similar. Furthermore, the three tests produced comparable estimates of predictive values. In an elimination setting, a serial interpretation of the results of RDT and LM should guarantee high NPV and PPV; attributes that are indispensable in assuring treatment efficiency and guiding surveillance activities geared towards eliminating falciparum malaria in primary care settings.

## Data availability

### Underlying data

The raw dataset for the study is stored under restricted access since it contains sensitive participant information. Notwithstanding, accessibility is possible upon placing a formal request to our Data Governance Committee ( dgc@kemri-wellcome.org). The replication data and analysis scripts for this manuscript are available from the Harvard Dataverse.

Harvard Dataverse: Replication data for: Bayesian evaluation of the performance of three diagnostic tests for *Plasmodium falciparum* infection in a low-transmission setting in Kilifi County, Kenya. https://doi.org/10.7910/DVN/Z5RBBT ^30^

This project contains the following underlying data:

- BLCM_malaria_tests_code.R (Bayesian Latent Class R code for malaria tests evaluation)
- Ngerenya_tests_data.tab (Study dataset)

Data are available under the terms of the Creative Commons Zero “No rights reserved” data waiver (CC0 1.0 Public domain dedication).
