# Supplementary material for: Bayesian evaluation of the performance of three diagnostic tests for Plasmodium falciparum infection in a low-transmission setting in Kilifi County, Kenya
Source: Wellcome Open Res. 2019 Oct 1;4:67. Originally published 2019 Apr 8. [Version 3] doi: 10.12688/wellcomeopenres.15204.3 (PMC6777009; doi:10.12688/wellcomeopenres.15204.3)
Supplement: Peer review history

# Peer review: Reviewer response for version 3

**DOI:** 10.21956/wellcomeopenres.16955.r36620

**Published:** 2019-10-02

**Authors:** Gillian Stresman

**Affiliations:** 1 Department of Infectious & Tropical Diseases, London School of Hygiene & Tropical Medicine, London, UK

I have no further comments.

I confirm that I have read this submission and believe that I have an appropriate level of expertise to confirm that it is of an acceptable scientific standard.

# Peer review: Reviewer response for version 2

**DOI:** 10.21956/wellcomeopenres.16839.r36218

**Published:** 2019-09-30

**Authors:** Linda Eva Amoah

**Affiliations:** 1 Noguchi Memorial Institute for Medical Research (NMIMR), University of Ghana, Accra, Ghana

Revisions have sufficiently addressed my concerns. I withdraw my reservations and would like to amend the status to Approved

I confirm that I have read this submission and believe that I have an appropriate level of expertise to confirm that it is of an acceptable scientific standard.

# Peer review: Reviewer response for version 2

**DOI:** 10.21956/wellcomeopenres.16839.r36217

**Published:** 2019-08-20

**Authors:** Gillian Stresman

**Affiliations:** 1 Department of Infectious & Tropical Diseases, London School of Hygiene & Tropical Medicine, London, UK

Thank you for taking the time to address my comments. The revised version of the paper is much improved. However, I still have one comment that I think merits some attention. Namely, the authors have carefully stressed that the population being studied is restricted to children. What is missing is that these are all 1) symptomatic/febrile and 2) care-seeking children. Although not all fevers are likely attributable to the malaria infection, any ones that are would typically be associated with higher parasite densities. Therefore it is not surprising that the performance of both RDT and LM tests was so good. This caveat with the population being studied is not made clear in the conclusions, both in the abstract and main text which is particularly important as it affects the generalisability of these results. For example, the performance of the diagnostic tests is good in symptomatic care-seeking children in this low transmission setting. This is a minor but important nuance.

I confirm that I have read this submission and believe that I have an appropriate level of expertise to confirm that it is of an acceptable scientific standard, however I have significant reservations, as outlined above.

# Peer review: Reviewer response for version 1

**DOI:** 10.21956/wellcomeopenres.16591.r35868

**Published:** 2019-07-22

**Authors:** Linda Eva Amoah

**Affiliations:** 1 Noguchi Memorial Institute for Medical Research (NMIMR), University of Ghana, Accra, Ghana

The main aim of the study was to estimate the sensitivity, specificity and predictive values for parasite prevalence estimated by light microscopy, HRP2 based RDT and PCR in a group of children.

Abstract:

- Methods: Only the total number of children recruited was stated and the exact number of asymptomatic vs symptomatic children was not stated. 
- Conclusions: LM and RDT tests RDT, rapid diagnostic tests ends with test so the test after RDT should be replaced or removed.

Introduction:

- The sentence ''the tests target...” is inaccurate, there are other Plasmodium antibodies on malaria RDT kits other than P. fal HRP2 and LDH.

Methods:

- The study comprises of "asymptomatic". This should be rephrased. The study comprised of healthy, asymptomatic and symptomatic children.
- The sample size for the three categories (healthy, asymptomatic and symptomatic) of children should be stated.
- How much blood was collected? The volume should be stated.
- What is the definition of fever? And what is fever status?
- The sample collection section only stated blood was collected and used for thick and thin smears and RDT preparation. Where did the blood for the PCR come from?
- Technically 30ul of blood being eluted with 100ul of buffer suggests very low DNA concentrations and in an asymptomatic population, a very low concentration. Could the negative PCR results just be the result of very low template concentrations?
- The description for the PCR procedure was very confusing, please rephrase with clarity. Was 5ul of DNA and blood both used for the amplifications?

Conclusion:

- No p-value in any of the tables were significant, thus this statement in the conclusion is very confusing and seemingly inaccurate.

I confirm that I have read this submission and believe that I have an appropriate level of expertise to confirm that it is of an acceptable scientific standard, however I have significant reservations, as outlined above.

# Peer review: Reviewer response for version 1

**DOI:** 10.21956/wellcomeopenres.16591.r35739

**Published:** 2019-06-25

**Authors:** Gillian Stresman

**Affiliations:** 1 Department of Infectious & Tropical Diseases, London School of Hygiene & Tropical Medicine, London, UK

**Overall comments:**

 This manuscript describes use of a latent class analysis to estimate the performance of three different diagnostic tools for malaria. The models and statistical methods are well described and the results are an interesting addition to the thinking about malaria diagnostic performance in a low transmission setting. However, I can only approve with reservations. As this paper is currently written, important nuances with the interaction between diagnostic test sensitivity and parasite densities (e.g. clinical infections vs infections identified in the community), sufficient details on the study population for the results to be interpreted accordingly, and how this relates to the broader generalizations that can be made should be addressed. 

**Specific comments:**

- The background argument to justify the study should be reframed slightly to better reflect the nuances with malaria diagnostics. The diagnostic performance of RDTs and LM has been assessed vs PCR in many research settings and is now considered as the gold standard, including some of the references cited by the authors. The text suggests that this is not the case with LM being the main comparator.
- Similarly, the authors state that PCR has presumed superior accuracy, but PCR is known to have superior sensitivity compared to RDTs and LM. The key question with PCR, as the authors state, being does that matter in a clinical/elimination setting? There is evidence that standard PCR methods are not perfect with the harder we look, the more parasites we’ll find. This argument should be included here as a better justification of the approach taken. 
- Samples are based on suspected clinical cases under 12 years of age and children 15 years of age sampled in the community. These populations are inherently different in terms of their likely parasite densities in infected individuals (with high parasitemia related to the probability of clinical symptoms) and demographically in terms of factors associated with care-seeking behavior. This is a critical strata that is currently missing to assess any effects on the Sp/Se of the tests and may contribute to the lack of effects observed. 
- As a related point, in the discussion, the authors state that Se/Sp of RDT and PCR have been found to be similar in the diagnosis of severe malaria in children in a clinical setting. This is not surprising as clinical cases of malaria are likely to have parasite densities well above the limit of detection of RDTs. This is very different than detection infections with expected low parasite densities. As above, the critical implications of the populations being studied on the interpretation of the results in this context should be more carefully expressed and acknowledged.
- Parasite densities in children are expected to be higher and more likely to be detectable using routine diagnostic tools (see Wu *et al.*, 2015 ^1^). It is therefore critical to interpret the resulting Se/Sp estimates as detection of malaria infections in children. Results from this study are not generalizable to adult populations.
- In the introduction the authors rightly claim that light microscopy is the standard for clinical malaria diagnosis and that its quality is highly variable. It might clarify these important nuances by distinguishing between expert microscopy, which has high Se and Sp, and routine microscopy, which is known to have a detection limit 10-fold higher than that stated and more similar to that of RDTs.
- I’m not aware of any RDTs for *Pf *that detect LDH only. Do the authors mean detect HRP-II *and *LDH? 
- In discussing RDTs it would be helpful to follow the same format as per microscopy and include the limit of detection for parasite densities for the routinely used tests, which is available via the WHO website.
- In terms of the definition of the ‘season’ variable, I’m not entirely clear about which population this is referring to. Is this for the clinical cases only? What time period of suspected cases were eligible for inclusion? In the methods section this is not clear. Similarly, for the community samples, the authors state that it’s a cross-sectional survey, which implies a single time point but also mention cohort, which implies multiple timepoints of sampling per individual. Was the community work part of a panel study where repeated cross-sectional surveys were conducted in the same population? Please clarify in both the methods and the definition of seasonality. 
- “In the present study setting, the prevalence of *P. falciparum *was estimated to be 5.6%...”  - what malaria metric was used to derive this estimate?
- The authors are correct in that HRP-II gene deletion in parasites will impact the Se, but so will circulating antigen levels that are below the limit of detection. It may also take time for antigen to build up to detectable levels, etc. There are lot of reasons why RDT Se is imperfect. A more thorough discussion would be helpful to highlight these important nuances. 
- False positive test results are equally of concern in high-prevalence settings (and arguably more likely given residual HRP-II).
- LDH tests are also much less sensitive in detecting infections so may not be the best choice for monitoring treatment efficacy.
- False positive PCR results may also be linked to contamination in the lab process.
- Although routine use of PCR in most malaria endemic settings is impractical as the authors suggest, in low transmission/pre-elimination settings would there be a role for reference labs to be set up for confirmatory testing?
- In the introduction the authors state that low density/PCR detectable infections are of unconfirmed importance, but the conclusion is that they are essential to remove as they are a potential source of onward transmission? This seems contradictory. In the introduction are the authors referring to the current WHO position? And in the conclusion the position from some of the research community?

I confirm that I have read this submission and believe that I have an appropriate level of expertise to confirm that it is of an acceptable scientific standard, however I have significant reservations, as outlined above.

## References

[1] : Comparison of diagnostics for the detection of asymptomatic Plasmodium falciparum infections to inform control and elimination strategies. *Nature*.2015;528(7580) : 10.1038/nature16039 S86-S93 10.1038/nature16039 26633770

# Response

**Published:** 2019-08-13

**Authors:** Marshal Mweu

**Affiliations:** University of Nairobi, Kenya

We are highly appreciative of your comments and suggestions towards improving the quality of this manuscript. However, there are a few issues you raise that we feel could be best addressed here:

1. **I’m not aware of any RDTs for Pf that detect LDH only. Do the authors mean detect HRP-II and LDH?**

**Response:** Some available literature does make specific mention of RDTs that detect only LDH – see for instance Wongsrichanalai et al. ^6^ and WHO ^9^.

2. **False positive test results are equally of concern in high-prevalence settings (and arguably more likely given residual HRP-II).**

**Response:** Going by PPVs, in a low-prevalence setting a test with a moderate Sp is likely to generate a much lower confidence in a positive result compared to using the same test in a high-prevalence location. Therefore, false positive results would, arguably, be of higher concern in the former settings.

3. **LDH tests are also much less sensitive in detecting infections so may not be the best choice for monitoring treatment efficacy.**

**Response:** Although in general LDH tests are less sensitive, when specific interest lies in monitoring treatment efficacy, a number of authors contend that Sp is a more desirable attribute ^6,9,47 ^– which disfavours HRP-II tests owing to antigen persistence.

4. **Although routine use of PCR in most malaria endemic settings is impractical as the authors suggest, in low transmission/pre-elimination settings would there be a role for reference labs to be set up for confirmatory testing?**

**Response:** Although, reference labs are indeed vital (and are actually in place) in elimination settings, we believe that a suitable confirmatory test should be one that affords a high Sp and thus PPV. For this reason a serial combination of available diagnostics (such as RDT and LM as proposed here) should assure an almost complete confidence in a positive test result.
